# Clinico-epidemiological and immunological characteristics of rickettsioses in a Sri Lankan patient cohort 2018–2023

**DOI:** 10.1186/s12879-025-10775-z

**Published:** 2025-03-19

**Authors:** Nayana Gunathilaka, Nilmini Chandrasena, Hemantha Sudusinghe, Vidusha Nethsara Mudalpath, Deshaka Jayakody, Ranjan Premaratna

**Affiliations:** 1 Department of Parasitology, Faculty of Medicine, University of Kelaniya, Ragama, Sri Lanka; 2 Department of Medicine, Faculty of Medicine, University of Kelaniya, Ragama, Sri Lanka

**Keywords:** Rickettsioses, Clinico-epidemiological, Immunofluorescence assay, Diagnosis

## Abstract

**Background:**

Rickettsioses, caused by intracellular bacteria of the genera *Rickettsia* and *Orientia*, are transmitted to humans through arthropod vectors such as ticks, fleas, and mites. Over the past two decades, this disease has been recognized as a significant cause of acute febrile illness in Sri Lanka. However, only a limited number of studies have focused on clinico-epidemiological characteristics of patients and immunological diagnostic approaches for disease confirmation.

**Method:**

A cross-sectional study was conducted at the Rickettsial Disease Diagnostic and Research Laboratory (RDDRL), University of Kelaniya, Sri Lanka, from 2018 to 2023 from the clinically suspected patients referred for disease confirmation. Clinical, demographic, epidemiological, biochemical, and laboratory data were collected via a questionnaire by reviewing the archived records. The serological finding of the immunofluorescence assay (IFA) conducted for patients samples were retrieved. The patients who were positive for IFA-IgG (> 1:128 as per presumptive confirmation of acute rickettsial illness) were taken as the test group and the negative group was taken as the control group. Data were analyzed using chi-square tests followed by a Correlation analysis between the variables using Pearson correlation.

**Results:**

Out of 1,221 cases, 249 (20.4%) were serologically “confirmed” as positive for rickettsial infection. The test group consisted predominantly of males, similar to the control group. Most cases were males and < 9 years of age followed by 10–19 years. Among the age groups, 10–19 years and 50–59 years, categories indicated a significant positive relationship according to the chi-squared statistics (*P* < 0.05). A seasonal trend was observed, with higher case numbers reported from January to February. Laboratory findings indicated significant differences between test and control groups in leucopenia (*P* = 0.005, χ²=7.87), increased neutrophil count (*P* = 0.0004, χ²=12.71), elevated alanine aminotransferase (*P* = 0.0001, χ²=14.64), elevated aspartate aminotransferase (*P* = 0.0001, χ²=18.24), urine occult blood (*P* = 0.024, χ²=5.09), and raised erythrocyte sedimentation rate (*P* = 0.034, χ²=4.51). Clinical manifestations showed no major deviations. Notably, eschar was more prevalent *in O. tsutsugamushi* cases (33.3%) compared to SFG rickettsioses (13.3%).

**Conclusion:**

This study highlights the need for improved awareness, diagnostic facilities, and vector control measures to manage rickettsial infections effectively in Sri Lanka. Understanding epidemiological patterns and clinical manifestations is crucial for developing effective surveillance and prevention strategies.

**Clinical trial:**

Not applicable.

**Supplementary Information:**

The online version contains supplementary material available at 10.1186/s12879-025-10775-z.

## Background

Rickettsioses are a group of infectious diseases caused by intracellular bacteria within the genera *Rickettsia* and *Orientia*, transmitted primarily through arthropod vectors such as ticks, fleas, and mites. These diseases are classified based on the species involved and their vectors. The main categories include the Spotted Fever Group (SFG), caused by *Rickettsia* species like *R. rickettsii*, which are transmitted by ticks; the Typhus Group, including *R. prowazekii* (epidemic typhus) and *R. typhi* (murine typhus), transmitted by lice and fleas; and the Coxiella Group, which includes *C. burnetii*, the causative agent of Q fever.

The Scrub Typhus Group includes *Orientia tsutsugamushi*, a distinct bacterium from *Rickettsia* species but still categorized within rickettsial infections due to its similar intracellular lifestyle and vector-borne transmission by chigger mites. Symptoms of rickettsioses generally include fever, headache, muscle pain, rash, and malaise, with more severe cases potentially leading to organ failure and death if untreated [1, 2]. While *Rickettsia* and *Orientia* species share key features, such as vector transmission and intracellular survival in host cells, *O. tsutsugamushi* differs from *Rickettsia* in terms of taxonomy, vector, and some clinical features. For example, *O. tsutsugamushi* is transmitted by chigger mites and causes scrub typhus, which may present with an eschar at the bite site, a feature not typically seen in *Rickettsia* infections [3].

In Sri Lanka, the earliest documented evidence of rickettsial infections traces back to the era spanning 1937–1945, coinciding with the period of the Second World War, marked by extensive military activities primarily concentrated in the Eastern Province [4]. Concurrently, this period also witnessed the detection of scrub typhus in the southern coastal areas of the island [5]. The disease is prevalent mainly in rural or suburban areas of the country. In general, the middle or lower socioeconomic classes in the rural/ suburban areas are at risk of exposure to wild and domestic animals and, therefore, the disease exposure acquisition [6]. The occupations involving outdoor activities predominantly represent the patients recorded thus far [7–9].

In Sri Lanka, rickettsioses have been increasingly recognized as an important cause of acute febrile illness, with several outbreaks reported in recent years [4, 7, 8]. At present, an average of 1,500 cases are notified to the Epidemiology Unit, Ministry of Health annually [10]. Although cases have been reported from all districts in Sri Lanka, the disease transmission is restricted to specific localities especially, Western, North-western and Northern provinces for chigger-mite-borne scrub typhus caused by *O. tsutsugamushi* and Central province for tick-borne Spotted Fever Group (SFG) typhus by SFG [4, 11] and a mixed pattern of SFG typhus, scrub typhus, and murine typhus caused by *R. typhi* in the Southern province [12].

Limited awareness of its prevalence and clinical detection among healthcare professionals are main challenges encountered in facing rickettsial infections in Sri Lanka [4, 13]. Due to similarities in clinical presentation with other common tropical febrile illnesses, such as dengue fever and leptospirosis, rickettsioses may often go undiagnosed or misdiagnosed, leading to delayed treatment and increased morbidity [7]. Since there is no readily available diagnostic facility in the public or private sector, disease confirmation has become challenging [14]. At present, two to three research centers in the country offer disease diagnoses that are driven through investigator-driven research grants and collaborations, but the population catered to such facility is limited [4]. The findings of these centers are limitedly available to the scientific community.

Epidemiological characteristics of scrub typhus in Sri Lanka is inadequately documented and the differences in clinical characteristics and temporal and regional distribution require further study. Owing to its variable and nonspecific clinical presentation, weak knowledge, low index of suspicion among doctors, and lack of diagnostic resources are key reasons for it being typically underdiagnosed of rickettsioses in Sri Lanka. Therefore, understanding the epidemiological patterns, clinical manifestations, and immunological responses associated with rickettsial infections is crucial for effective disease management, surveillance, and implementation of preventive strategies in Sri Lanka.

## Method

### Study design and setting

Rickettsial Disease Diagnostic and Research Laboratory (RDDRL), Faculty of Medicine, University of Kelaniya, Sri Lanka was established in 2008 in collaboration with CDC Atlanta, Georgia, USA. This laboratory provides a disease diagnostic facility to patients who are clinically suspected of having rickettsial infection. They are referred from various public and private sector hospitals island-wide. The laboratory maintains a database consisting of clinical details and laboratory investigation results which are provided in a detailed structured request-form that should accompany the appropriate blood or serum samples at the time the rickettsial disease diagnostic tests were requested. A cross-sectional study was conducted by recruiting all patients referred to the RDDRL, of the Faculty of Medicine, University of Kelaniya, Sri Lanka for rickettsial disease diagnosis from 2018 to 2023.

### Case definition and diagnostic criteria

Clinical parameters and diagnostic criteria used as clinical characteristics common to rickettsial infection in previously published studies were used to recruit participants for the present study. Patients with a high intermittent fever (≥ 38.5 °C to 40 °C) and at least five out of the following eight clinical features were included in the study: headache, myalgia, regional lymphadenopathy, generalized lymphadenopathy, hepatomegaly, splenomegaly, presence of an eschar, or presence of a maculopapular rash, and rapid defervescence with doxycycline or chloramphenicol [6, 11, 12].

### Collection of primary data

Clinical, demographic, epidemiological, biochemical and other laboratory investigation information that were recorded on formatted data sheets were retrieved for the present investigation.

### Laboratory indicators

The laboratory indicators analyzed in this study included routine blood tests, biochemical markers, and urinalysis, along with additional diagnostic assessments such as erythrocyte sedimentation rate (ESR), C-reactive protein (CRP), chest X-rays, and electrocardiograms (ECG). Complete blood count (CBC) parameters included leukopenia (WBC < 4 × 10⁹/L), leukocytosis (WBC > 10 × 10⁹/L), neutrophilia (neutrophils > 75%), and thrombocytopenia (PLT < 100 × 10⁹/L). Biochemical markers assessed were liver function tests (ALT > 40 U/L and AST > 40 U/L) and inflammatory markers, including CRP (> 10 mg/L) and ESR (elevated if > 15 mm/h in males and > 20 mm/h in females). Urinalysis included the detection of occult blood and proteinuria. Additional diagnostic assessments included abnormal chest X-rays (e.g., infiltrates or pleural effusions) and ECG findings indicating conduction abnormalities or arrhythmias. These laboratory parameters were evaluated to determine their association with serologically confirmed rickettsial infections and to identify key hematological and biochemical trends in suspected cases.

### Serological testing for rickettsial antibodies

Blood samples (4 ml) were collected from clinically suspected patients between the 5th and 7th day of illness and stored at -20 °C until testing. Serological testing was conducted using Indirect Immunofluorescence Assay (IFA), established at the Rickettsial Disease Diagnostic and Research Laboratory (RDDRL), Faculty of Medicine, University of Kelaniya, Sri Lanka. The test used lyophilized antigen pellets targeting *Rickettsia conorii* (Malish) for Spotted Fever Group (SFG) and *Orientia tsutsugamushi* (Karp, Gilliam, Kato) for Scrub Typhus Group (STG). The same IFA test was used to detect IgG antibodies against both *Rickettsia* and *Orientia* species. Serum samples were diluted in phosphate-buffered saline (PBS) before testing, and each sample was tested in duplicate to ensure consistency.

Fluorescein-conjugated goat anti-human IgG and IgM antibodies were used for detection, and slides were examined under a fluorescence microscope by two independent investigators. Positive controls included previously confirmed IFA-positive sera, while negative controls consisted of serum from healthy individuals with no history of rickettsial exposure. As this was not a commercially certified IFA test, the cut-off value for presumptive confirmation of acute rickettsial illness was established at IgG titre ≥ 1:128, based on international guidelines and previous studies in Sri Lanka [4]. These measures ensured the reliability of IFA testing for diagnosing rickettsial infections.

### Data analysis

All the data were entered into a Microsoft Excel worksheet and all the data analysis was performed using GraphPad Prism (Version 10.2.0). Descriptive statistics (number and percentages were calculated. Data were analyzed using chi-square tests to determine the association between socio-demographic, laboratory/investigation results, clinical signs & symptoms in test and control groups. Correlation analysis between the variables in the test and control groups was performed using Pearson correlation. Further, the association between SFG and *O. tsutsugamushi* infections relevant to the above aspects was also analyzed using the Chi-square test. The *P* value of 0.05 (*P* < 0.05) was considered as statistically significant. Temporal and seasonal distribution of rickettsia positive (Test: T) and negative (Control: C) by the IFA test was elucidated by a heat map generated using GraphPad Prism version 10.2.1 for Windows (GraphPad Software, San Diego, CA, USA, www.graphpad.com) with monthly number of cases recorded in each.

## Results

### Socio-demographic characterization of test and control groups

**Fig. 1:**
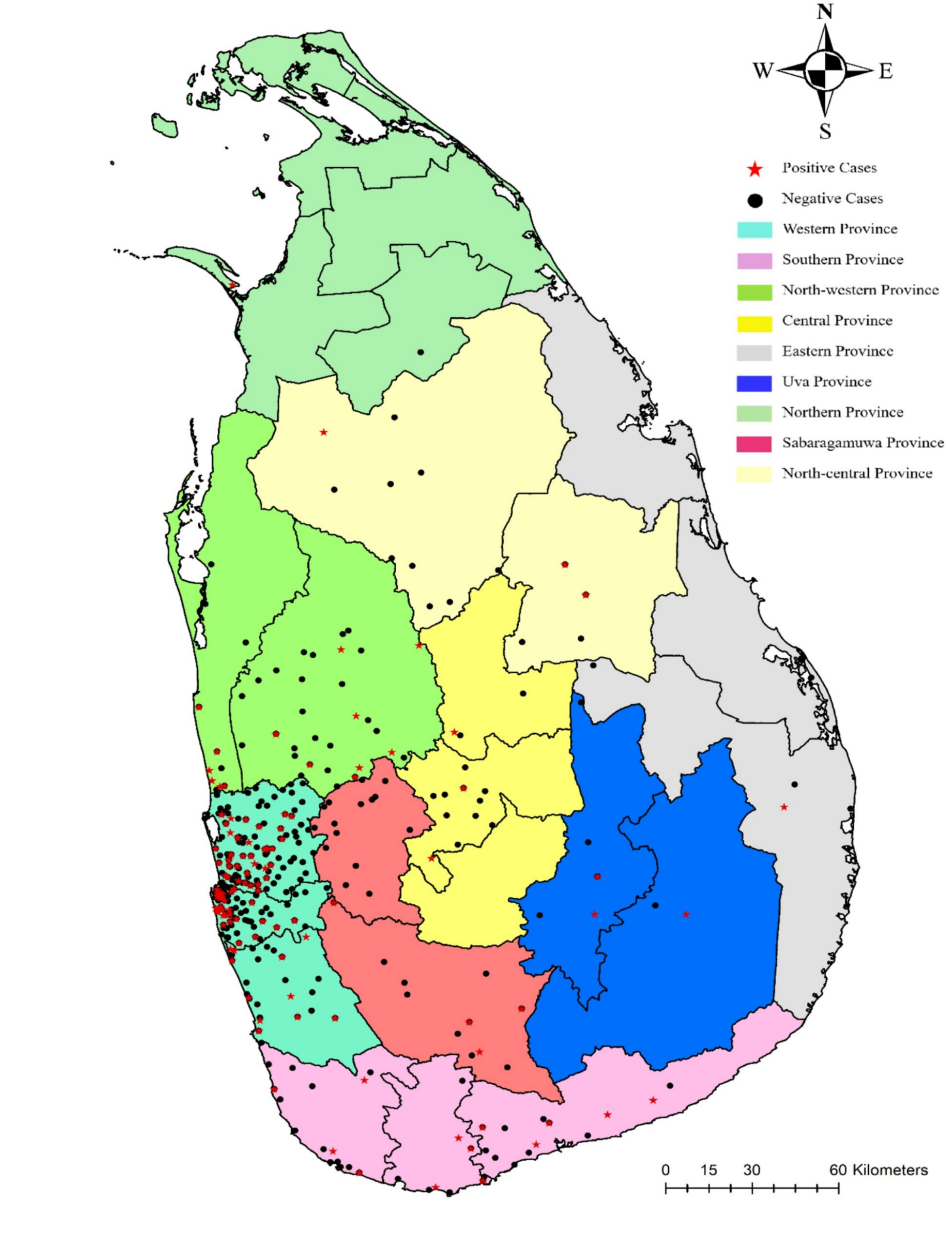
A Sri Lankan map indicating clinically suspected positive and negative cases of rickettsial infections in each province during 2019–2023

A total of 1221 cases that are clinically suspected of rickettsiosis have been reported for disease diagnosis from 2018 to 2023. Of them, 249 patients have been serologically confirmed by IFA (20.4%) and 972 patients have become negative (79.6%). Therefore, IFA positive cohort was taken as the test group (T) and the negative group was considered as the control group (C). In both test and control groups, males were predominant (Table 1). The majority of the clinically suspected cases were reported from western province mainly from Gampaha and Colombo Districts. The distribution of clinically suspected negative and positive cases is indicated in Fig. 1. In both test and control groups, age < 9 years comprised the majority (positive: 20.48%; negative: 19.75), followed by 10–19 years (positive:10.84%; negative: 16.26%). Among the age groups 10–19 years and 50–59 years, categories indicated a significant positive relationship according to the chi-squared statistics (*P* < 0.05).

**Table 1:** Socio-demographic characteristics test and control groups considered in the study

Factor	Test group		Control group		Chi-square	95% CI	*P* valve
	*n*	%	*n*	%			
**Gender**							
Male	142	57.03	526	54.12	0.679	0.670–1.181	0.41
Female	107	42.97	446	45.88			
**Age group**							
< 9	51	20.48	192	19.75	0.133	0.668–1.333	0.715
10–19	27	10.84	158	16.26	4.179	1.014–2.417	0.0409*
20–29	26	10.44	119	12.24	0.502	0.752–1.843	0.478
30–39	33	13.25	118	12.14	0.227	0.600 -1.377	0.634
40–49	32	12.85	104	10.7	1.088	0.530–1.210	0.297
50–59	45	18.07	129	13.27	4.119	0.468–0.984	0.0424*
60–69	24	9.64	105	10.8	0.213	0.707–1.756	0.644
70–79	10	4.02	42	4.32	0.028	0.529–2.181	0.866
> 80	1	0.4	5	0.51	0.045	0.175–14.94	0.831
**Occupation**							
Pre-school	32	12.85	107	11.01	0.668	0.548–1.275	0.414
Student	48	19.28	207	21.3	0.489	0.803–1.599	0.484
Unemployed	22	8.84	61	6.28	2.05	0.422–1.129	0.152
Farmer	8	3.21	15	1.54	2.99	0.207–1.067	0.084
Manual laborer	3	1.2	10	1.03	0.058	0.252–2.910	0.809
Military services	4	1.61	11	1.13	0.368	0.218–2.024	0.544
Business/ Trade	1	0.4	19	1.95	2.968	0.893–51.90	0.085
Private sector employers	20	8.03	87	8.95	0.209	0.682–1.871	0.647
Government sector employers	5	2.01	34	3.5	1.423	0.694–4.224	0.233
Non responders	106	42.57	421	43.31	0.045	0.775–1.368	0.833
**Residence**							
Western Province	199	79.92	793	81.58	0.361	0.782–1.57	0.548
North-western Province	14	5.62	53	5.45	0.011	0.541–1.77	0.916
Southern Province	14	5.62	40	4.12	1.065	0.383–1.31	0.302
Sabaragamuwa Province	5	2.01	38	3.91	2.109	0.794–4.72	0.146
North-central Province	9	3.61	18	1.85	2.848	0.228–1.16	0.092
Central Province	3	1.20	9	0.93	0.158	0.212–2.65	0.691
Uva Province	3	1.20	8	0.82	0.324	0.204–2.39	0.569
Eastern Province	1	0.40	8	0.82	0.481	0.320–22.95	0.488
Northern Province	1	0.40	5	0.51	0.052	0.177–15.17	0.820

* Significant at *P* < 0.05

### Information of the laboratory investigations

Laboratory investigation results in all clinically suspected patients are shown in Table 2. Only 249 patients were serologically positive for rickettsial infection (20.4%). Most of them (*n* = 166; 66. 67%) were positive for SFG followed by *O. tsutsugamushi* (30.12%; *n* = 75) and mixed infections (3.12%; *n* = 8). Chi-square statistics conducted for test and control groups indicated significant differences with leucopenia (*P* = 0.005; *χ*^*2*^ = 7.87), increased neutrophil count *(P* = 0.0004; *χ*^*2*^ = 12.71), elevated alanine aminotransferase (ALT) (*P* = 0.0001; *χ*^*2*^ = 14.64), elevated aspartate aminotransferase (AST) (*P* = 0.0001; *χ*^*2*^ = 18.24) urine occult blood (*P* = 0.024; *χ*^*2*^ = 5.09), and erythrocyte sedimentation rate (ESR) (*P* = 0.034; *χ*^*2*^ = 4.51).

**Table 2:** Comparison of the investigation results of patients in test and control groups

Laboratory investigation	Test Group *n* (%)	Control Group *n* (%)	Chi-square	*P*-value
**Haematological parameters**				
Leucopenia (WBC < 4 × 10^9/L)	13 (5.22)	62 (6.38)	7.87	0.005*
Leukocytosis (WBC > 10 × 10^9/L)	82 (32.93)	345 (35.49)	2.57	0.109
Neutrophil increased (*N* > 75%)	40 (16.06)	207 (21.30)	12.71	0.0004*
Thrombocytopenia (PLT < 100 × 10^9/L)	19 (7.63)	84(8.64)	0.74	0.390
**Biochemical parameters**				
Alanine aminotransferase increased (ALT > 40U/L)	95 (38.15)	271 (27.88)	14.64	< 0.0001*
Aspartate aminotransferase increased (AST > 40U/L)	103 (41.37)	289 (29.73)	18.24	< 0.0001*
**Urinalysis**				
Urine Red cells	48 (19.28)	218 (22.43)	5.09	0.024*
Urine protein	24 (9.64)	84 (8.64)	0.54	0.462
**Inflammation index**				
C reactive protein (CRP > 10 mg/L)	138 (55.42)	543 (55.86)	0.17	0.681
ESR (male > 15 mm/h, female > 20 mm/h)	86 (34.54)	366 (37.65)	4.51	0.034*
Abnormal chest X-Ray	30 (12.05)	104 (10.70)	2.42	0.120
Abnormal ECG	10 (4.02)	34 (3.50)	0.89	0.346
**Immunofluorescence assay**				
*Orientia tsutsugamushi*	75 (30.12)			
*Rickettsia conorii*	166 (66.67)			
Mixed infections	8 (3.21)			

* Significant at *P* < 0.05

The Pearson correlation coefficient indicated a linear relationship between two variables. Strong positive correlations were observed when leukocytosis with increased neutrophils (*r* = 0.1420), elevated ALT and elevated AST level; increased ALT with increased AST (*r* = 0.7651), urine red cells, and increased CRP (*r* = 0.2126); elevated AST with Urine RBC (*r* = 0.2597) and increased CRP (*r* = 0.2126; urine RBC with urine protein (*r* = 0.5861). Based on the chi-square test results, there is no significant association between the type of rickettsial infection (*O. tsutsugamushi* vs. SFG) and the laboratory investigation results. This suggests that the laboratory findings are not differentially influenced by the type of rickettsial infection in this dataset (Table 3).

**Table 3:** Comparison of the investigation results of patients positive for *Orientia tsutsugamushi* and SFG by Immunofluorescence assay

Investigation	Orientia tsutsugamushi *n*(%)	SFG *n*(%)	Chi-square value	*P*- value
**Haematological parameters**				
Leucopenia (WBC < 4 × 10^9/L)	6 (8.00)	6 (3.61)	1.569	0.210
Leukocytosis (WBC > 10 × 10^9/L)	29 (38.67)	50 (30.12)	2.377	0.123
Neutrophil increased (*N* > 75%)	10 (13.33)	28 (16.87)	0.786	0.375
Thrombocytopenia (PLT < 100*10^9/L)	6 (8.00)	12 (7.23)	0.065	0.799
**Biochemical parameters**				
ALT increased (ALT > 40U/L)	32 (42.67)	60 (36.14)	1.092	0.296
AST increased (AST > 40U/L)	36 (48.00)	64 (38.55)	2.365	0.124
**Urinalysis**				
Urine RBC	14 (18.67)	32 (19.28)	0.054	0.816
Urine protein	4 (5.33)	19 (11.45)	1.941	0.163
**Inflammation index**				
CRP increased (CRP > 10 mg/L)	37 (49.33)	97 (58.43)	2.429	0.119
ESR (male > 15 mm/h, female > 20 mm/h)	30 (40.00)	74 (44.58)	0.701	0.402
**Other**				
Abnormal chest X-Ray	8 (10.67)	20 (12.05)	0.172	0.678
Abnormal ECG	2 (2.67)	6 (3.61)	0.233	0.630

### Temporal and seasonal distribution of cases

**Fig. 2:**
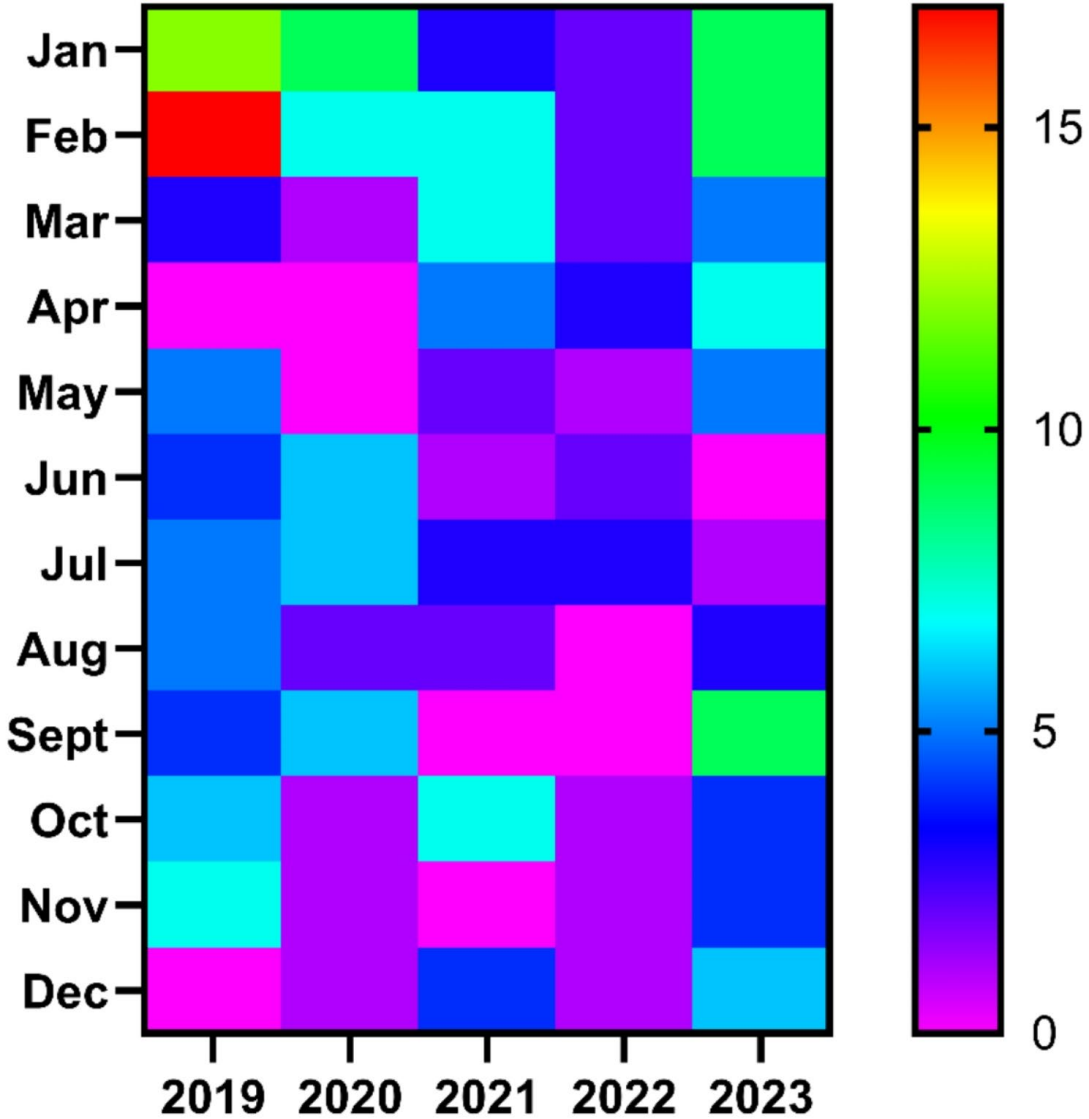
Monthly distribution of rickettsioses positive cases reported to Rickettsial Disease Diagnostic and Research Laboratory (RDDRL), Faculty of Medicine, University of Kelaniya, Sri Lanka from 2019–2023

The heat map statistics of positive cases are illustrated in Fig. 2. A notable seasonal trend in the clinical illness reporting cases was observed among the test group. January and February showed relatively higher case incidences, as evidenced by the warmer colours (red and yellow) during these months across several years. This trend suggests that the early part of the year is a peak period for rickettsial infections (Fig. 2). However, there is variability in the number of cases reported each year. For example, February 2019 shows a particularly high incidence (red), while other years like 2020 and 2021 have more moderate levels of cases during the same period. Certain months, such as July and August, consistently showed lower incidences of cases (represented by blue and purple colors). This suggests a seasonal low during mid-year, possibly due to climatic factors or reduced vector activity. The map highlights a significant increase in cases in November 2023, which stands out as an anomaly compared to previous years. This peak could indicate an outbreak or increased transmission during this specific period.

### Signs and symptoms of patients

Clinical manifestations among positive and negative groups had no major deviations. The majority of them had fever, headache and body aches as main complaints (Table 4). It was observed that among positive cases, the presence of rash (23.7%; *n* = 59) and eschar (15.7%; *n* = 39) was higher although not significant. Patients infected with *O. tsutsugamushi* and with symptoms of SFG is shown in Table 5. The symptoms such as fever, headache, and joint pains were the major complaints in both disease types. However, body aches in *O. tsutsugamushi* infections were higher (50.7%; *n* = 38) compared to SFG (39.8%; *n* = 66). In addition, a higher percentage of patients with SFG had a cough (31.9%; *n* = 53) as a clinical manifestation than in *O. tsutsugamushi* infected patients (16%; *n* = 12). In terms of signs, the majority of the patients that were positive for SFG indicated rash as a main presentation (28.3%; *n* = 47) while the patients with *O. tsutsugamushi* indicated 13.3% (*n* = 10). Further, the presence of an eschar was higher in *O. tsutsugamushi* patients (33.3%; *n* = 25) and only 13.33% was observed among *SFG* patients.

Overall, the presence of rash (*P* = 0.0005) and eschar (*P* = 0.0001) among these two infections was statistically significant according to the chi-square statistics. Lymphadenopathy was comparatively higher among *O. tsutsugamushi* cases (25.3%; *n* = 19) although not statistically significant (Table 5). Rash (*P* = 0.010; *χ*^*2*^ = 6.69), eschar (*P* = 0.002; *χ*^*2*^ = 9.39), and lymphadenopathy (*P* = 0.043; *χ*^*2*^ = 4.10) show significant associations between positive cases and negative cases. Cough (*P* = 0.008; *χ*^*2*^ = 7.09), rash (*P* = 0.009; *χ*^*2*^ = 6.85), and eschar (*P* = 0.001; *χ*^*2*^ = 10.87) showed significant associations between *O. tsutsugamushi* and SFG infections.

**Table 4:** Comparison of clinical signs and symptoms in test and control groups used in the study

Symptoms & Signs	Positive Cases *n* (%)	Negative Cases *n* (%)	Chi-square value	*P*-value
**Symptoms**				
Fever	192 (77.11)	749 (77.06)	0.000	0.990
Headache	110 (44.18)	464 (47.74)	1.463	0.226
Body aches	107 (42.97)	359 (36.93)	3.448	0.063*
Joint pains	73 (29.32)	254 (26.13)	1.258	0.262
Cough	69 (27.71)	284 (29.22)	0.311	0.577
Confusion	12 (4.82)	51 (5.25)	0.09	0.765
Fits	5 (2.01)	24 (2.47)	0.074	0.785
Neck stiff	7 (2.81)	23 (2.37)	0.034	0.853
Diarrhea	35 (14.06)	112 (11.52)	1.596	0.206
Psychiatric manifestations	8 (3.21)	29 (2.98)	0.032	0.857
Tinnitus	1 (0.40)	9 (0.93)	0.253	0.615
Deafness	0 (0.00)	3 (0.31)	-	-
**Signs**				
Rash	59 (23.69)	175 (18.00)	6.696	0.010*
Eschar	39 (15.66)	94 (9.67)	9.388	0.002**
Lymphadenopathy	47 (18.88)	136 (13.99)	4.104	0.043*
Papilledema	0 (0.00)	16 (1.65)	-	-
Conjunctival Hemorrhages	0 (0.00)	5 (0.51)	-	-
Exudates	0 (0.00)	3 (0.31)	-	-
Pulse rate > 100	40 (16.06)	145 (14.92)	0.211	0.646
Blood pressure > 120/80	34 (13.65)	144 (14.81)	0.154	0.695

** Significant at *P* < 0.05; * Significant at *P* < 0.1

**Table 5:** Comparison of signs and symptoms among *Orientia tsutsugamushi* and *Rickettsia conorii* (*SFG*) detected from this study

Symptoms & Signs	Orientia tsutsugamushi *n* (%)	Rickettsia conorii (SFG) *n* (%)	Chi-square Value	*P*-value
**Symptoms**				
Fever	57 (76.00)	128 (77.11)	0.052	0.819
Headache	34 (45.33)	71 (42.77)	0.189	0.664
Body aches	38 (50.67)	66 (39.76)	2.085	0.149
Joint pains	23 (30.67)	47 (28.31)	0.168	0.682
Cough	12 (16.00)	53 (31.93)	7.092	0.008 **
Confusion	2 (2.67)	10 (6.02)	2.276	0.131
Fits	1 (1.33)	4 (2.41)	0.38	0.538
Neck stiff	1 (1.33)	6 (3.61)	1.232	0.267
Diarrhea	11 (14.67)	21 (12.65)	0.227	0.634
Psychiatric manifestations	4 (5.33)	4 (2.41)	1.105	0.294
Tinnitus	0 (-)	1 (0.60)	0.376	0.54
**Signs**				
Rash	10 (13.33)	47 (28.31)	6.849	0.009 **
Eschar	2 (33.33)	13 (7.83)	10.87	0.001 ***
Lymphadenopathy	19 (25.33)	27 (16.27)	2.066	0.151
Pulse rate > 100	12 (16.00)	28 (16.87)	0.068	0.794
Blood pressure > 120/80	13 (17.33)	21 (12.65)	1.221	0.269

** Significant at *P* < 0.01; * Significant at *P* < 0.05

### Distribution of eschar in the body

**Fig. 3:**
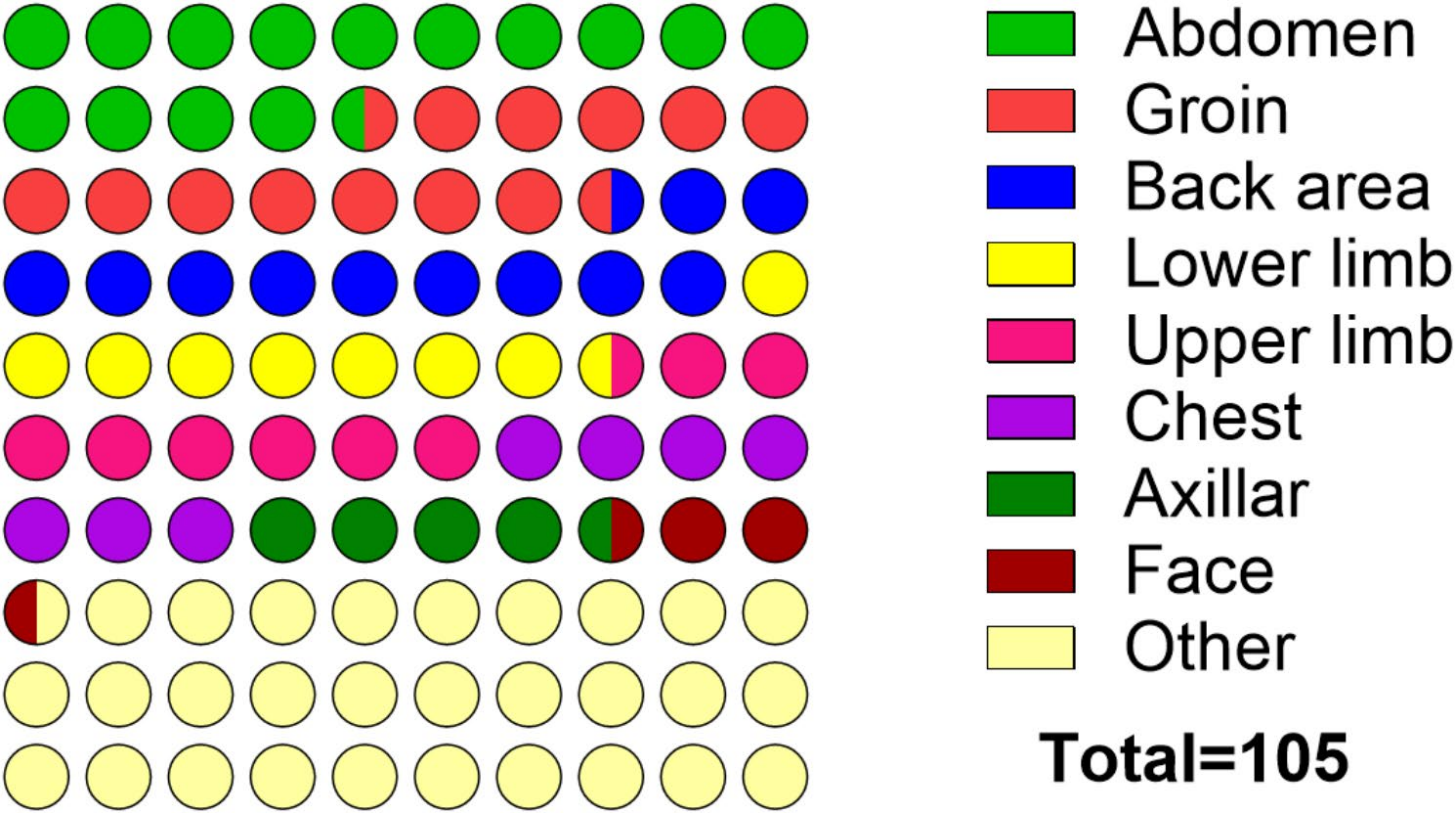
The distribution of eschar in 105 suspected patients, negative for Rickettsioses (Control)

The characteristic eschar was found in 134 clinically suspected cases of scrub typhus (Positive: *n* = 29; Negative: *n* = 105) out of 1221 patients (percentage prevalence: 10.9%). A few patients indicated multiple eschar lesions. Of the control cases, the eschar was identified mainly from other areas in the body (29.52%) which include the breast, penis, scalp, perianal, etc., followed by the abdomen (14.29%), groin (13.33%) and back area (11.43%) (Fig. 3).

**Fig. 4:**
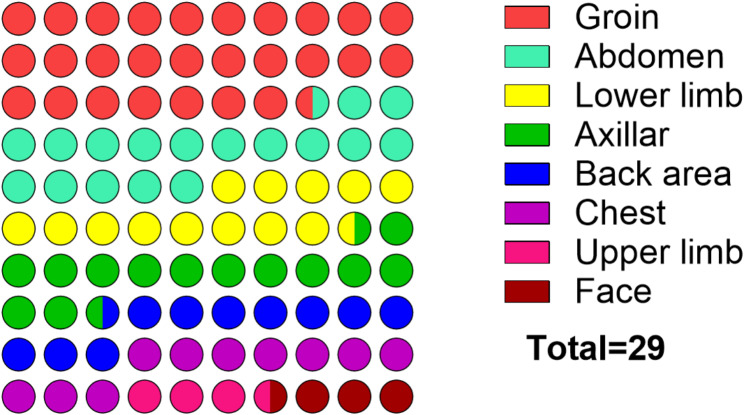
The distribution of eschar in 29 suspected patients that were positive for Rickettsioses

Of the 29 patients that were serologically positive indicated eschars mainly in the groin (27.59%), abdomen (17.24%), lower limb (13.79%), and axillar (13.79%) (Fig. 4).

## Discussion

The present study is likely to provide a comprehensive further knowledge analysis of rickettsial infections in Sri Lanka, aligning with previous literature while also highlighting and adding new insights on epidemiological trends and clinical manifestations. The findings underscore the significant public health challenge posed by rickettsioses, particularly in rural and suburban areas of Sri Lanka. The current study reaffirms these trends, with the majority of cases reported from Western, North-Western, and Northern provinces, consistent with previous reports [4, 6].

The predominance of males in the serologically confirmed cases (20.23%) from 2019 to 2023 is consistent with previous studies conducted in Sri Lanka. Research by Premaratna et al. [15] and Kularatne et al. [7] also reported a higher incidence in males, likely to be due to occupational exposure to vectors in outdoor activities. This trend is similarly observed in studies conducted in China [16]. Previous studies conducted locally and elsewhere have highlighted predominance among 40–69 years of age which was related to occupation related field activities such as farming [16–18]. However, alarmingly, the present study highlight higher incidence in younger age groups < 9 years followed by the 10-19-year category indicating significant positive relationships in the age group between 10 and 19 years. The reason for such change could well be due to underutilization of empiric anti-rickettsial antibiotics such as doxycycline in paediatric age group by first contact general practitioners. However, this observation needs further evaluation to arrive at conclusions. The majority of study samples were from the western province and that could be due to close access to the diagnostic facility which is established in the same province. Although rickettsioses is considered a rural disease, the disease burden in urban areas may not be as low as assumed [16]. In general, the western province of Sri Lanka is considered urban than rural. However, it has many agricultural land, and cater for many outdoor recreational outdoor activities, such as walking, gardening and sports. Therefore, the eco-friendly trend of having more natural parkland and gardens within the city may create more suitable habitats for vectors and rodent hosts and ultimately increase the exposure risk [18].

The seasonality of the cases indicates that the occurrence of cases was linked with the temporal and seasonal patterns [19]. In general, ambient temperature, humidity, rainfall and availability of food during the harvesting are linked with the rodent survival which ultimately favours disease transmission [16, 20]. The seasonality observed in the temporal distribution of cases, with peaks in January-February and November, aligns with patterns reported in other regions, likely influenced by climatic conditions affecting vector activity [7, 21]. This seasonality highlights the need for targeted public health interventions during these high-risk periods.

The study reveals that fever, headache, and body aches to be predominant symptoms in both positive and negative cases, similar to findings from earlier studies [7]. However, the presence of rash and eschar, while more common in positive cases, did not show significant difference compared to controls, most likely due to their non-specific nature. Furthermore, we had no facilities to investigate these patient samples for *R. typhi* infection where fever, an eschar or rash is expected. However, within rickettsial diseases the finding of rash and eschars are in line with research from Thailand and India [22].

In the clinical spectrum, *O. tsutsugamushi* infections were associated with more body aches and eschar, whereas *SFG* infections were more likely to present with a rash and cough. These differences are statistically significant and may help in differential diagnosis. The characteristic eschar distribution among positive cases, primarily located in the groin, abdomen, and lower limbs, supports earlier observations [23–24]. The presence of an eschar although not specific, may suggest rickettsial disease more likely. Studies have documented atypical eschar sites in scrub typhus, which can often be overlooked and lead to misdiagnosis or mixed infections, a challenge noted in past literature [7]. These atypical sites are noted to occur in warm, damp areas of the body where skin surfaces meet or clothes bind, such as the perineum, groin, axilla, and underneath the breast [25–26]. Eschar locations often correspond to areas of tight clothing or skin folds, probably because such sites prevent further ascend of the vector along the body and thus vector attachment for its feed [27].

Interestingly, cough was more prevalent in patients with *R. conorii* infection (31.9%) compared to those with *O. tsutsugamushi* (16%). This distinction could be crucial for differential diagnosis, as similar studies in Korea have noted respiratory symptoms more commonly in spotted fever group rickettsiosis compared to scrub typhus [28]. Presence of acute onset cough and high fever may easily carry a misdiagnosis of respiratory tract infection [29]. Furthermore, lymphadenopathy was more common in *O. tsutsugamushi* cases (25.3%), although not statistically significant, which aligns with findings from Southeast Asia where lymphadenopathy is a notable feature of scrub typhus [30]. Therefore, limited awareness and recognition of rickettsial infections, nonspecific nature of clinical symptoms similar to other febrile illnesses such as dengue and leptospirosis together with absence of readily available confirmatory diagnostic facilities often leads to misdiagnosis of these illnesses as reported in previous studies [7].

In laboratory investigations, significant differences were observed for leucopenia, increased neutrophil count, elevated ALT, elevated AST, Urine Occult blood and ESR for rickettsial infections compared to non-rickettsial illness similar to documented in previous studies [31]. For example, elevated liver enzymes (ALT and AST) are well-documented in cases of rickettsial diseases, indicating hepatic involvement [31]. The significant correlations between leukocytosis, increased neutrophils, elevated ALT, and AST levels further support the systemic inflammatory response associated with these infections. These laboratory results suggested that rickettsial infection could affect multiple system functions, including the blood system, liver function, kidney function, coagulation function, and various inflammatory indicators.

It is important to note that, out of 1,221 clinically suspected cases, only 249 (20.4%) were serologically confirmed, indicating approximately four-fifths of clinically suspected cases were due to other illnesses. This highlights the need to improve the clinical knowledge and diagnostic skills of local physicians to enhance the accuracy of rickettsial disease suspicion, particularly in resource-limited settings. Given the limited availability of confirmatory serological testing in Sri Lanka, training programs should focus on equipping healthcare workers with the ability to recognize rickettsioses based on clinical signs, epidemiological patterns, and basic laboratory markers. Additionally, the establishment of diagnostic facilities in major regional hospitals could facilitate timely and accurate disease confirmation, reducing misdiagnosis and unnecessary antibiotic use. Findings from this study could contribute to the development of clinical guidelines to assist in differentiating rickettsial infections from other febrile illnesses, ultimately improving patient outcomes.

This study has several limitations inherent to its retrospective design. Since data collection was based on patient records and laboratory databases that were not originally intended for research purposes, some information may be incomplete or missing. Selection bias may have influenced the findings, as the study population consisted of patients referred to a single diagnostic centre, potentially excluding undiagnosed or unreported cases from other healthcare settings. Furthermore, serological testing based on IFA, while specific, may not detect all cases, particularly in the early stages of infection or in patients with low antibody titers. Seasonal and temporal trends were inferred from heat maps, which, while useful, do not provide a precise measure of incidence over time. Lastly, the study did not account for potential confounding factors such as co-infections or underlying health conditions that could influence clinical manifestations and laboratory results. These limitations highlight the need for further longitudinal studies with larger and more diverse populations to validate and expand upon these findings. Despite of all these limitations, this study emphasizes the need for continuous surveillance, early diagnosis, and targeted interventions to manage and control rickettsial diseases effectively.

## Conclusion

The findings indicate that males and young children are more commonly affected, with significant geographic clustering in the Western Province according to the reported cases. Laboratory investigations revealed significant associations with leucopenia, increased neutrophil count, elevated liver enzymes (ALT and AST), urine occult blood, and ESR, suggesting these parameters are useful markers for rickettsial infections. Clinical manifestations such as fever, headache, body aches, rash, and eschar were predominant among confirmed cases, with notable differences between infections caused by SFG and *O. tsutsugamushi*. The study also identifies seasonal patterns, with higher case reports in the early months of the year, underscoring the importance of temporal surveillance in managing outbreaks. Despite limitations, these findings contribute valuable insights for the diagnosis and management of rickettsial infections, emphasizing the need for continued surveillance and research to mitigate the public health impact of these diseases in Sri Lanka.

## Electronic supplementary material

Below is the link to the electronic supplementary material.

**Supplementary Material 1**: The questionnaire used to receive clinico-epidemiological and immunological characteristics of rickettsioses clinically suspected patients.

## Data Availability

Data is provided within the manuscript.

## Abbreviations

CRP: C-reactive protein
ESR: Erythrocyte sedimentation
IFA: Immunofluorescence assay
RDDRL: Rickettsial disease diagnostic and research laboratory
SFG: Spotted fever group
