# Supplementary material for: Clinico-epidemiological and immunological characteristics of rickettsioses in a Sri Lankan patient cohort 2018–2023
Source: BMC Infect Dis. 2025 Mar 19;25:379. doi: 10.1186/s12879-025-10775-z (PMC11921605; doi:10.1186/s12879-025-10775-z)
Supplement: Supplementary file 1 — Supplementary Material 1: The questionnaire used to receive clinico-epidemiological and immunological characteristics of rickettsioses clinically suspected patients. [file 12879_2025_10775_MOESM1_ESM.pdf]

Supplementary material 1. The questionnaire used to receive clinico-epidemiological and immunological characteristics of rickettsioses clinically suspected patients

Request for IFA assays for suspected rickettsioses

Institution:..Ward:..........................BHT:.............Date:..........................

Consultant:..Contact No:...........................H/Officer:..........................

Patient Name:..Age:...............M/F

Address:...District:..............................

Occupation: ....Contact No: (Home).........................(Mobile).....................

Fever Duration:................................days, Highest:......................Frequency:......................./day

Headache: Y/ N; Frontal / Occipital / Patrietal Body aches: Neck/ Arms/ Legs/Back

Joint pains: Small Jts [Small] Jt (R/ L) Feet (R / L)] Wrist (R/ L) / Elbow (R/ L) /

Shoulder (R/L) / Ankle (R / L) / Knee (R/ L) / Hips (R / L) / Spine: Cx /Lumber

Cough: Y/N SOB: Y/N. Confusion: Y/N Fits: YIN Neck stiff Y/N

Rash: Y/N (Maculo-papular / Fern Leaf): UL / LL, Thorax / Back / Face /Palms / Soles)

Diarrhea: Y/N if Yes: Date of onset in relation to fever:..

Any psychiatric manifestation: Y/N if yes:..

Eschar: Y/N Site:...Number:..........................

LN: Cx/Axilla/Inguinal: other:..L: Y/N:..............cm SP: Y/N

Pulse:........................BP:................/................mmHg Tinnitus: Y/N, Deafness: Y/N

Fundoscopy: Papillodema: Y/L, Haemorrhages: Y/N, Exudates: Y/N, Eshcar Bx: Y/N

WBC:......................./mm^3 : N.......% L:............. ESR:.............1st Hr, CRP:.............iu/L

Platelet:....................SGPT:...........Iu/L, SGOT:............Iu/L UFR: RBC:..........................

Protein:..ECG:...

Chest X ray:...

Other:...

Contact: Rats/Dogs/Cattle/other:.........................Sleep: Floor/Scrub land/Sand

Recent visits:..................................Tick Exposure: Y/N
